# Risk factors for psychotic relapse in chronic schizophrenia after dose-reduction or discontinuation of antipsychotics. A systematic review and meta-analysis

**DOI:** 10.1192/j.eurpsy.2021.1426

**Published:** 2021-08-13

**Authors:** J. Bogers, G. Hambarian, J. Vermeulen, L. Dehaan

**Affiliations:** 1 High Care Clinics, MHO Rivierduinen, Leiden, Netherlands; 2 Psychiatry, Amsterdam University Medical Centre, Amsterdam, Netherlands

**Keywords:** dose reduction, Relapse, Risk factors, meta-analysis

## Abstract

**Introduction:**

Patients are often treated with high doses or combinations of antipsychotics, which may hamper recovery. Dose-reduction (DR) or discontinuation of antipsychotic medication in chronic patients carries the risk of psychotic relapse.

**Objectives:**

To identify risk factors of psychotic relapse after DR or discontinuation, we (i) determined the rate of relapse after DR or discontinuation in patients with chronic schizophrenia, and (ii) assessed risk factors for psychotic relapse.

**Methods:**

From studies on dose-reduction from January 1950 through June 2019 we calculated event rates per person-years including 95% confidence intervals. We extracted: (1) patient characteristics (age, percentage of male subjects, setting, duration of illness), (2) dose-reduction/discontinuation characteristics (start-dose, end-dose, dose-reduction in milligrams and percentage of start-dose, time-period of dose-reduction), (3) follow-up characteristics (time after dose-reduction), and (4) study characteristics (blinding, publication-year and relapse definition).

**Results:**

46 unique cohorts, presenting 1677 patients in which doses were reduced/discontinued were included in meta-analysis. We found an overall event rate per person-years on psychotic relapse of 0.55 (CI95% 0.46-0.65;p<0.0001;I^2^ =79). Most robust event rates for psychotic relapse were seen for discontinuing antipsychotics, and if not discontinuing, dose-reduction till under 5mg haloperidol equivalents daily (HE). Abrupt reduction yielded higher rates than gradual reduction. During short follow-up time more relapses occurred than in studies with long follow-up time.

**Conclusions:**

In patients with chronic schizophrenia discontinuing, and to a lesser extent DR till end-dose<5mgHE, patients who reduce doses abrupt, inpatients, and patients with a short duration of illness carry highest relapse risk. Most relapses occur during the first half year after DR.

